# Nutrigonometry III: curvature, area and differences between performance landscapes

**DOI:** 10.1098/rsos.221326

**Published:** 2022-11-30

**Authors:** Juliano Morimoto, Pedro Conceição, Knut Smoczyk

**Affiliations:** ^1^ Institute of Mathematics, University of Aberdeen, King’s College, Aberdeen AB24 3FX, UK; ^2^ School of Biological Sciences, University of Aberdeen, Zoology Building, Tillydrone Avenue, Aberdeen AB24 2TZ, UK; ^3^ Programa de Pós-graduação em Ecologia e Conservação, Universidade Federal do Paraná, Curitiba 82590-300, Brazil; ^4^ Institute of Differential Geometry, Riemann Centre for Geometry and Physics, Welfengarten 1, Hannover 30167, Germany

**Keywords:** ecological specialization, Grinnellian niche, diet, climate change, persistence homology

## Abstract

Nutrition is one of the underlying factors necessary for the expression of life-histories and fitness across the tree of life. In recent decades, the geometric framework (GF) has become a powerful framework to obtain biological insights through the construction of multidimensional performance landscapes. However, to date, many properties of these multidimensional landscapes have remained inaccessible due to our lack of mathematical and statistical frameworks for GF analysis. This has limited our ability to understand, describe and estimate parameters which may contain useful biological information from GF multidimensional performance landscapes. Here, we propose a new model to investigate the curvature of GF multidimensional landscapes by calculating the parameters from differential geometry known as Gaussian and mean curvatures. We also estimate the surface area of multidimensional performance landscapes as a way to measure landscape deviations from flat. We applied the models to a landmark dataset in the field, where we also validate the assumptions required for the calculations of curvature. In particular, we showed that linear models perform as well as other models used in GF data, enabling landscapes to be approximated by quadratic polynomials. We then introduced the Hausdorff distance as a metric to compare the similarity of multidimensional landscapes.

## 1. Introduction

Animals can often balance their nutrient intake to maximize fitness [1,2]. This creates the potential for nutritional trade-offs to emerge when animals cannot simultaneously maximize the nutrient balance for the expression of two competing fitness-related traits. [3–5]. Nutritional trade-offs are ubiquitous and have been described across the animal kingdom, from invertebrates such as flies [6–8] and crickets [9,10] to vertebrates [11–13], including humans [14,15]. Nutritional trade-offs shape an individual’s fitness and can have important implications for responses to unbalanced diets as well as adaptations to novel environments [16,17].

Nutrition is complex and the ability of individuals to navigate nutritional trade-offs and properly balance their nutrient intake depend both on the amount of—and the (synergistic and antagonistic) interactions between—nutrients [5]. A recent model known as the geometric framework (GF) enables the complexity of nutrition to be studied in relatively simple experimental designs, where both the quantity and the interactions between nutrients can be investigated simultaneously [18,19]. GF has gained central stage in studies of nutrition both in animals and humans and has underpinned major insights into the evolution of nutritional responses (e.g. protein leverage hypothesis [20]) [4,5]. Despite this, the development of analytical frameworks to analyse properties of GF multidimensional performance landscapes has lagged behind, and many studies have relied on visual interpretations to draw conclusions (see e.g. [21–24]; but see also [25]). While the visual approach can be useful for simple inferences, it is neither objective nor reproducible. More importantly, it overlooks the nuanced properties of multidimensional landscapes which might contain useful biological information about the responses of animals to nutrition.

Recent models have been developed to automate and standardize the analysis of GF performance landscapes. For example, Rapkin *et al.* [26] proposed the use of the coefficients of regression models, obtained by regressing the performance trait *i* to the intake of nutrients (say *P* and *C* for protein and carbohydrate, respectively) as components of vectors $v_{i}$ for each performance trait *i*. This enables the comparison between the angle *θ*_*i*,*j*_ of vectors $v_{i}$ and $v_{j}$ as proxy of the strength of nutritional trade-offs. We showed that the use of coefficients could lead to inaccurate estimates of the angle *θ*_*i*,*j*_ [27]. Instead, we proposed the use of the positions of the coordinates representing the region of interest in the performance landscapes of traits *i* and *j* as the components of the (position) vector $v_{i}$ and $v_{j}$, from which the nutritional trade-off can be more accurately estimated from the angle *θ*_*i*,*j*_. Other models have been proposed to find peaks and valleys in multidimensional performance landscapes, using either bootstrapping [28] or machine learning models [27]. More recently, we also proposed a novel way to define the peaks and valleys of multidimensional performance landscapes for comparison of strengths of nutritional trade-offs using the angle *θ*_*i*,*j*_ which strictly represents performance landscapes as right-angle triangles and uses trigonometry for estimates of nutritional trade-offs [29]. However, these models focus on obtaining information on either peak or valley regions (or both) of the multidimensional performance landscapes, overlooking other properties of the landscapes with potential biological significance.

Here, we explored this and proposed a model to calculate both the surface-area and local curvatures of multidimensional performance landscapes. These two properties of performance landscapes can be extracted and compared against the expected value of a flat landscape, and thus can provide invaluable information as to the overall profile of the nutritional responses not only in the regions of optimal (peaks) and minimal (valleys) responses, but across the entire sampling space of nutrients. We refer to performance landscape sensu [27] in that the landscape represents the possible values of the performance trait (e.g. lifespan) across a range of (*x*, *y*) values. Importantly, animals do not necessarily ‘walk’ onto the performance landscapes. Hence, performance landscapes can be thought of as a ‘blueprint’ for the expression of a performance trait. This means that the definition of performance landscapes is different than the definition of fitness landscapes sensu stricto, the latter of which incorporates fitness, phenotype and genotype [30]. We first explain the mathematics underpinning the estimates of curvatures in multidimensional landscapes. For this, we assumed that multidimensional performance landscapes can be approximated by a quadratic polynomial of the form *ax*^2^ + *by*^2^ + *cx* + *dy* + *exy*, where *a*, *b*, *c*, *d*, *e* are the coefficients of a general linear model (LM) and *x*, *y* correspond to protein and carbohydrate intakes, respectively. We then describe how the surface-area of multidimensional performance landscapes can be estimated and compared against the area of a flat landscape of the same region, which provides a proxy of how wiggly the landscape is relative to a flat landscape. Next, we demonstrated the application of the model in canonical datasets (flat landscape and a saddle landscape) and to a landmark GF dataset which investigates nutritional responses in terms of lifespan, lifetime egg production (‘lifetime eggs’) and daily egg production (‘daily eggs’) in *Drosophila melanogaster*. To do this, we first tested the assumption that GF landscapes can be approximated by a quadratic polynomial by comparing the performance of a quadratic polynomial regression (LM), a generalized additive model (GAM) and a thin-plate spline (TPS) model, the latter two of which are commonly used to analyse GF data. We then calculated surface-area and the Gauss and mean curvatures of the performance landscapes for lifespan, lifetime eggs and daily eggs. Finally, we compared the performance landscapes against a flat landscape as well as against each other using a metric known as the Hausdorff distance, which enabled us to compare two multidimensional performance landscapes of *n*-dimensions. This has the potential to expand the tools in which two landscapes can be directly compared, broadening our ability to make inferences about nutritional trade-offs when two performance landscapes are substantially different. Overall, the model proposed here advances our ability to study the properties of multidimensional performance landscapes and can underpin important biological insights from multidimensional studies in nutritional ecology and evolutionary biology.

## 2. The model

The model was designed to estimate properties of performance landscapes that have so far been overlooked. This is because GF studies and models have primarily focused on identifying regions of maxima or minima in the performance landscape, that is, peaks and valleys. While useful, this approach might neglect other properties of the performance landscapes which may contain important information to characterize the landscapes and gain insights into the biological responses to varying nutrient balances. To discuss the model, we first present the Taylor’s theorem and the use of polynomial approximations to describe the performance landscapes. Next, we provide an intuition of curvature and present the Theorema Egregium. We then focus on the application of these two concepts to performance landscapes of empirical work to demonstrate how the model presented here can be used. For the purpose of the discussion and for the target audience of the paper (e.g. biologists), we denote an affine subspace with zero curvature as a ‘flat surface’.

### 2.1. Taylor’s theorem and polynomial approximation of performance landscapes

Given any smooth function *f* (or at least *k*-differentiable), it is possible to apply Taylor expansion to obtain a polynomial approximation of *f* of any degree *k* around a given point—this is known as the *Taylor’s theorem* [31]. The resulting polynomial is called the *k*th-order Taylor polynomial. It is important to note that to compute curvature, the smooth function *f* must be at least two-differentiable (i.e. differentiable at least up to the second degree). This is because the first derivative *f*′ of the smooth function *f* provides the slope of the tangent line or plane while the second derivative *f*″ provides information about the concavity of the tangent line or plane, and hence, its curvature. Taylor’s theorem can be applied to more than one variable, providing a way to approximate functions with *n* variables using *k* degree polynomials. The approximated function is *k*-differentiable and can be used to estimate curvature of the landscape when *k* ≥ 2. For the purpose of this study and the available empirical data in the literature, we will focus on functions with two variables (*x*, *y*) and approximations using *k* = 2 (i.e. quadratic approximations). Nonetheless, the arguments used here are applicable to functions with *n* variables and *k* degree polynomials.

### 2.2. Intrinsic and extrinsic curvature and the Theorema Egregium

As mentioned above, let’s consider only smooth functions and in particular, performance landscapes approximated by a polynomial of degree 2. Curvature at each point *p* is defined via *principal curvatures*. The two principal curvatures *κ*_1_ and *κ*_2_ represent, respectively, the maximum and minimum amount of ‘bending’ in the landscape in each orthogonal direction of movement in the landscape along the tangent plane (i.e. a plane tangent to a point *p* on the landscape). The principal curvatures are algebraically defined as follows: *κ*_1_ and *κ*_2_ (at a point *p*) are the eigenvalues of a linear operator (i.e. can be seen as a matrix) called the *shape operator*. The shape operator at point *p* is defined as

$$S(v)=\nabla_{v}(n)$$

where *v* is a vector tangent to *p*, $\nabla_{v}$ is the directional derivative and *n* is the unit normal vector field of a surface *M*. As $\nabla_{v}(n)$ is still a tangent vector at *p*, the shape operator is a linear operator

$$S_{p}\;:\;T_{p}M\to T_{p}M,$$

where *T*_*p*_*M* is the tangent space at a point *p* of *M*, that is, a vector space that encapsulates all the possible directions which pass tangentially through *p*. This makes sense because the eigenvectors measure the distortion of a linear operator, in this case, the eigenvalues of the shape operator tell us how much the landscape bends.

Principal curvatures bear relationship with *extrinsic* and *intrinsic* curvatures. Extrinsic curvature is a property of the landscape which depends on the space in which the landscape exists. In other words, extrinsic curvatures are properties that depend on the *embedding* of the landscape. Conversely, intrinsic curvature is a property that only depends on the landscape itself, that is, the intrinsic curvature depends only on the *metric space*.

The relationships between principal curvatures and extrinsic and intrinsic curvatures are simple and useful for analysing landscapes. For instance, the *mean curvature*, which is an extrinsic curvature, is the arithmetic mean of the principal curvatures, that is, (*κ*_1_ + *κ*_2_)/2. The *Gauss curvature*, an intrinsic curvature, is the product of the principal curvatures *κ*_1_*κ*_2_. Therefore, the Gauss curvature is the determinant of the shape operator and the mean curvature is the arithmetic mean of the trace of the shape operator. From linear algebra, the determinant of a matrix is invariant under change of bases (and row columns operations)—that is the intrinsic property—whereas the sum mean of the trace of the shape operator does depend on the embedding of the surface and, therefore, an extrinsic property.

**Figure 1.**
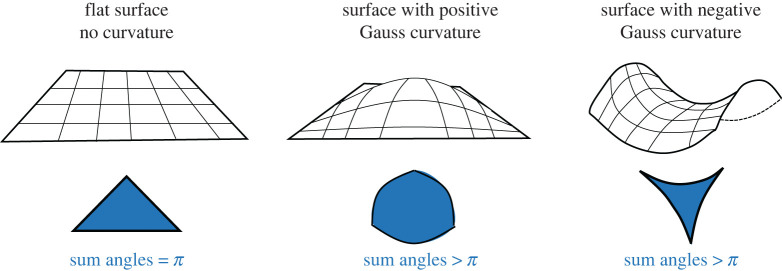
Schematic of the concept of curvature flat surfaces had no curvature such that, if we were to draw a triangle embedded on the surface, the sum of the internal angles would equal *π*. Surfaces or sub-sections of surfaces with positive Gauss curvature are curved in a way that, if we were to draw a triangle embedded in the surface, the sum of the internal angles would exceed *π*. This could be equivalent to sub-sections of the surface corresponding to the surface peak regions. Surfaces with negative Gauss curvature are curved in a way that, if we were to draw a triangle embedded on the surface, the sum of the internal angles would be less than *π*. This is equivalent to sub-sections of the surface corresponding to mountain pass regions. Note that, for the purposes of this paper, we use ‘flat surface’ to denote an affine subspace with zero curvature.

Importantly, the Gauss curvature (but *not* the mean curvature), as it is an intrinsic invariant, does not change value if the surface bends without stretching, that is, invariant under local isometries—this is known as Gauss’s *Theorema Egregium*. A consequence of this theorem illustrates the local to global interplay of geometry and topology. The theorem provides information as to when two spaces are different, for instance, a sphere of radius *r* has Gauss curvature 1/*r*^2^ and a plane has Gauss curvature 0. One way of developing an intuition about Gauss curvature in particular is to play with triangles. From Euclidean geometry, we know that the sum of the internal angles in any triangle sums up to *π*, that is, 180°. Geometric objects that satisfy this condition are called planar. But what if this condition fails? For instance, imagine that, instead of drawing a triangle in a flat piece of paper, we draw the triangle in the surface of a sphere. Note that the triangle is not allowed to leave the surface, that is, it must be drawn onto the surface itself. In this case, the sum of the internal angles of the triangle will be *greater* than *π*. If we were to compare the triangles drawn on a piece of paper and in the surface of a sphere, the latter is more *curved* than the former. Sphere-like spaces are known as *convex* surface or *(locally) positively curved spaces*, whereas flat-like spaces are referred to as spaces with *zero curvature*. There are also situations where the sum of the internal angles of a triangle is *less than* *π*, for example, a triangle embedded in a saddle surface. These are known as (locally) hyperbolic spaces or *(locally) negatively curved*. But what does this have to do with Gauss curvature? The sign of the Gauss curvature provides a way to identify the characteristics of the surface: a surface with zero Gauss curvature can be classified as flat. Surfaces with Gauss curvature positive and negative are classified as convex or hyperbolic, respectively (see figure 1). For the landscapes in nutritional ecology (and evolutionary biology, more generally), Gauss curvature can thus be an important parameter to characterize the local properties of the space that the landscape generates.

We can also obtain information on the local magnitude of the curvature of a given space that is positively or negatively curved. For example, as mentioned above, a flat plane has Gaussian curvature 0 across its entire domain, whereas a sphere of radius *r* has Gaussian curvature 1/*r*^2^ [32]. This means that, for a sphere, the larger its radius, the smaller the local curvature at a given point in the surface of that sphere. In other words, if you were to stand on top and walk along a perfectly spherical ball (in any direction, since this is a perfect sphere), it is easy to imagine that this is a much harder task than walking on the surface of the Earth. This is because the radius *r*_*s*_ of the football is substantially smaller than the radius *r*_*E*_ of the Earth, and thus, the local curvature of the former is greater than the local curvature of the latter (ask the Flat Earth Society!).

For practical applications, however, the Gauss curvature has limitations. For instance, imagine rolling a sheet of paper to form a cylinder. The flat sheet of paper has Gauss curvature equal to zero and, because there were no deformations such as stretching of the sheet of paper when rolling, the Gauss curvature of a cylinder is also zero. This happens because the cylinder is an isometric transformation of a flat plane (i.e. the transformation does not tear, stretch or shrink the flat surface). As a result, the Gauss curvature alone is unable to differentiate between the two forms. Yet, they are different, and we need additional metrics to differentiate them. This leads to the definition of other types of curvature which depend on properties of the object as well as the space in which the object exists (*extrinsic* properties of a surface). In this instance, we can resort to the estimates of the *mean curvature* which is non-zero for the cylinder but zero for a flat surface (see also [32] for formal definitions). Together, Gauss and mean curvatures provide important properties to characterize surfaces and, in our case, landscapes.

### 2.3. Curvature and nutrition

In nutritional ecology and GF experiments, the idea of curvature can be useful to understand how animals are expected to navigate nutrient imbalances given the shape of the performance landscape. For instance, animals navigating a performance landscape with small or zero curvature likely benefit (or pay costs) of nutrient imbalances in equal magnitude throughout the landscape, as they move from valley to peak regions. It is analogous to walking uphill (or around the hill, depending on the direction). This suggests that an imbalanced diet has the same relative cost to performance in two arbitrary regions of the landscape. Conversely, animals navigating a performance landscape with large curvature likely pay a greater relative cost to performance in certain regions of the landscape (e.g. on the edges) where the negative effect of nutrient imbalance is accelerated. Thus, understanding the curvature of a performance landscape can enable us to understand and predict the costs associated with nutrient imbalance.

Following the idea of curvature, we also propose to use the idea of *surface area* of the performance landscape as an additional metric for landscape characterization. The concept of area is easily understood and thus, we will not delve into analogies. One important point worth mentioning here is that surface-area allows us to estimate how ‘wiggly’ (rugged) a performance landscape is. This is because the wiggliness of a landscape increases its surface-area (analogous to e.g. cell microvilli) relative to a flat landscape with the same domain (i.e. values of *x* and *y*). In nutritional ecology and GF experiments, the wiggliness of the performance landscape can indicate how resilient the animal is to small changes in nutrient balance, and how plastic the animal is in maximizing performance traits in response to varying combinations of diet (e.g. the term we call ‘nutritional plasticity’). Performance landscapes with large surface-area likely represent cases where small deviations in nutrient balance of the diet create large differences in the expression of a performance trait (i.e. the landscape is wiggly). This could indicate that the performance trait responds rapidly to changes in nutrition and hence, is nutritionally plastic, but not resilient. On the other hand, performance traits with landscapes that are less wiggly indicate high resilience against deviations from nutrient balance, but potentially low nutritional plasticity.

With this intuition, we now present the formal mathematics underpinning the calculations of curvature and surface-area, with focus on the performance landscapes of interest to this study. Next, we apply the formulations to canonical and real-world datasets to demonstrate their use and interpretation.

### 2.4. Curvature of performance landscapes

Suppose *S* is a performance landscape parameterized as (x,y)↦(x,y,f (x,y)) where *f*(*x*, *y*) is given by a quadratic polynomial of the form *ax*^2^ + *by*^2^ + *cx* + *dy* + *exy* and *a*, *b*, *c*, *d*, *e* are coefficients of a general linear regression. The variables *x* and *y* can represent intakes of protein and carbohydrate, respectively. The domain of *f*(*x*, *y*) lies within [0, x*], [0, y*], where (x*, y*) are the maximum intake of nutrients *x* and *y* nutrients. This domain is identical for any *S*_1_, *S*_2_, …, *S*_*i*_, multidimensional performance landscapes obtained from the same experiment. For reasons that we discuss below, it is necessary to ensure that the domains of the multidimensional landscapes coincide. Therefore, to compare multidimensional performance landscapes from different experimental designs, the domains can be min–max standardized as (*x* − min (*x*))/(max (*x*) − min (*x*)) (and similarly for *y*) such that the domains of the landscapes are *x* = [0, 1] and *y* = [0, 1]. We can then estimate the Gauss curvature $K_{S_{i}}$ and mean curvature $H_{S_{i}}$ of a performance landscape *S*_*i*_. To do this, we first estimate the gradient $\nabla S_{i}$ in the *x*- and *y*-direction:

2.1 $$\nabla S_{x}=\frac{\partial S_{i}}{\partial x}=\left(\begin{array}{c} 1\\ 0\\ 2ax+c+ey \end{array}\right)\;\text{and}\;\nabla S_{y}=\frac{\partial S_{i}}{\partial y}=\left(\begin{array}{c} 0\\ 1\\ 2by+d+ex \end{array}\right)$$

Note that these partial derivatives provide the coordinates of tangent vectors to a point within the landscape in the (*x*, *y*) directions. As a result, we can also calculate the unit normal vector $\vec{n}$ perpendicular to the landscape at a given point as the cross product of the two partial tangent vectors in the directions of *x* and *y* such that

2.2 $$\vec{n}=\frac{\left(\partial S_{i}/\partial x)\times (\partial S_{i}/\partial y\right)}{\left\|(\partial S_{i}/\partial x)\times (\partial S_{i}/\partial y)\right\|}$$

Next, we calculate the second partial derivatives of $\nabla^{2}S_{i}$ as

2.3 $$\nabla^{2}S_{x}=\frac{\partial^{2}S_{i}}{\partial x^{2}}=\left(\begin{array}{c} 0\\ 0\\ 2a \end{array}\right)\;\text{and}\;\nabla^{2}S_{y}=\frac{\partial^{2}S_{i}}{\partial y^{2}}=\left(\begin{array}{c} 0\\ 0\\ 2b \end{array}\right).$$

We can now use the I and II fundamental forms to estimate Gauss and mean curvature at all points in the landscape. A simplified geometric intuition for the I and II fundamental forms can be obtained as follows: (i) the I fundamental form can be thought of as providing information on the curvature in the landscape for any direction of travel, starting at a given point in the performance landscape and (ii) the II fundamental form provides information on how much the landscape curves away (or deviates) from a flat tangent plane at a given point in the landscape. Note that the I fundamental form can be estimated solely using properties of the landscapes itself (and hence is related to ‘intrinsic’ curvature of the landscape) while the II fundamental form requires an additional parameter (i.e. the normal vector n) and hence provides ‘extrinsic’ curvature of the landscape. We calculate the I fundamental form as

2.4 $$E=\frac{\partial S_{i}}{\partial x}\cdot \frac{\partial S_{i}}{\partial x};\;F=\frac{\partial S_{i}}{\partial x}\cdot \frac{\partial S_{i}}{\partial y}\;\text{and}\;G=\frac{\partial S_{i}}{\partial y}\cdot \frac{\partial S_{i}}{\partial y}$$

and

2.5 $$I=\left(\begin{array}{cc} E & F\\ F & G \end{array}\right)$$

Likewise, we calculate the II fundamental form as

2.6 $$L=\frac{\partial^{2}S_{i}}{\partial x^{2}}\cdot \vec{n};\;M=\frac{\partial^{2}S_{i}}{\partial x\partial y}\cdot \vec{n}\;\text{and}\;N=\frac{\partial^{2}S_{i}}{\partial y^{2}}\cdot \vec{n}$$

and

2.7 $$II=\left(\begin{array}{cc} L & M\\ M & N \end{array}\right)$$

The Gauss curvature $K_{S_{i}}$ and the mean curvature $H_{S_{i}}$ can then be calculated as

2.8 $$K_{S_{i}}=\frac{LN-M^{2}}{EG-F^{2}}$$

and

2.9 $$H_{S_{i}}=\frac{GL-2FM+EN}{2(EG-F^{2})}$$

$K_{S_{i}}$ and $H_{S_{i}}$ provide information about the local curvature of the multidimensional performance landscapes. For biological purposes, this curvature contains information on the shape of the landscape as well as the local changes in shape of the landscape across valleys and peaks (see below). Importantly, these quantities provide information regarding additional properties of the GF multidimensional performance landscapes which, combined with previous methods, can collectively describe the regions as well as the overall shapes of the landscapes.

### 2.5. Area of performance landscapes

Multidimensional performance landscapes are not always flat. Instead, performance landscapes can have oscillations in the *z*-direction such that the surface area $A_{S_{i}}$ varies according to the magnitude of the oscillations. For performance landscapes that contain high degrees of oscillation (i.e. high *‘wiggliness’*), the surface-area is expected to be higher than that of flat performance landscapes if the two landscapes have the same domain. We then calculated the surface-area of performance landscapes as a proxy of how ‘wiggly’ a landscape is relative to the area of a flat landscape over the same domain. To do this, we can estimate the area of the flat surface *A*_0_ as the area of a rectangle with sides *x*, *y*. To estimate the surface-area $A_{S_{i}}$ of the performance landscapes, we can use the formula for the surface integral

2.10 $$A_{S_{i}}=\iint_{\Omega }\sqrt{1+(\nabla S_{i}{)}^{2}}\;\text{d}x\;\text{d}y=\iint_{\Omega }\sqrt{1+{\frac{\partial S_{i}}{\partial x}}^{2}+{\frac{\partial S_{i}}{\partial y}}^{2}}\;\text{d}x\;\text{d}y.$$

The double-integral is then evaluated over the intervals [0, *y*] and [0, *x*], respectively, to return the surface-area of the landscapes over its domain Ω. For performance landscapes in nutritional geometry, where we can consider *x* as protein intake and *y* as carbohydrate intake, we evaluate the surface integral from 0 to the maximum intake of *x* and *y* (max *x*, *y*) so that the equation above becomes

2.11 $$A_{S_{i}}=\int_{0}^{\max y}\int_{0}^{\max x}\sqrt{1+{\frac{\partial S_{i}}{\partial x}}^{2}+{\frac{\partial S_{i}}{\partial y}}^{2}}\;\text{d}x\;\text{d}y.$$

Alone, this metric is of little use. For a more informative estimate, we can calculate the ratio of the surface-area of the performance landscape over the surface-area of a flat landscape *S*_0_ with the same domain as the performance landscape. In doing so, we obtain the *surface-area ratio* of performance landscape *i* as $A_{i}=A_{S_{i}}/A_{0}$ where *A*_*i*_ = [1, ∞). The greater the value of *A*_*i*_, the greater the surface-area of the performance landscape relative to a flat landscape of the same domain. Conversely, performance landscapes with *A*_*i*_ closer to 1 are nearly flat. Thus, the surface-area ratio could be interpreted as the magnitude of wiggliness of a landscape, provided that the ratio informs us how many times the area of the performance landscape is greater than that of a similar flat landscape. Note that for estimating surface-area, for which the ratio is performed against a flat landscape with similar domain (as opposed to direct comparisons between two performance landscapes), the *z* value was maintained in its original scale to represent the deviations from a flat surface in the *z* dimension (but see also the section on Hausdorff distance below).

## 3. Material and methods

### 3.1. Statistical analyses

All analyses were conducted in R v. 4.1.3 [33]. Data handling was conducted using the tidyverse packages ‘dplyr’ and ‘tidyr’ [34]. Performance landscape plots and mean curvature plots were done using the ‘ggplot2’ package [35]. Closed solutions for the surface integrals and the function to estimate the Hausdorff distance were obtained from the ‘pracma’ package [36]. We estimated confidence intervals for the Hausdorff distance using the ‘boot’ function from the package of the same name [37].

We first validated our assumption that performance landscapes could be approximated using a quadratic polynomial. To do this, we split the data into training and testing sets in the proportion 60–40%. We then generated new datasets by sampling with replacement from the training set, and fitted one of three models: (i) general LM with trait value as response variable, the linear and quadratic effects of protein and cabrohydrate intakes, and the linear interaction between protein and carbohydrate intakes; (ii) generalized linear model (GAM) using the ‘gam’ package [38] with similar structure as the model above and with smoothing function ‘*s*’ with default parameters and (iii) a TPS model using the ‘fields’ package [39] with trait value as response variable and protein and carbohydrate intakes as independent variables. We estimated model performance using the square-root of the sum of the squared residuals (i.e. root-mean-square-error or RMSE) for both the training and testing datasets.

We then developed the algorithms to estimate curvature and surface-area. The underlying algorithm used in all analyses is as follows:

1. We fitted a general LM for each performance trait, with the trait value (simulated or empirical) as a dependent variable and a polynomial with the main linear and quadratic effects of protein and carbohydrate intakes and the linear interaction between protein and carbohydrate intakes.
2. We created a square grid from 0 to max protein and carbohydrate intakes, from which the predicted value of a point *x*, *y* corresponding to the intakes of protein and carbohydrate, respectively, could be predicted using the general LMs from above. This approach has two benefits:
  — It creates an interpolation similar to that obtained with *splines* but with the advantage of conforming to a generalized equation *ax*^2^ + *by*^2^ + *cx* + *dy* + *exy*. This polynomial facilitates integration and estimates of partial derivatives for calculation of curvature.
  — It ensures that the predicted values for all performance traits are obtained for similar regions within the domain of the performance landscape. This is because the models predict the expected value of each performance landscape using the same underlying *x*, *y* grid.
3. In addition to the predicted values for all traits, we included the values for a flat landscape of the form (*x*, *y*, 1). Note that the choice of 1 was arbitrary and does not affect curvature or surface-area.
4. We then used the grid containing the predicted values for each trait to estimate curvature, surface-area and Hausdsorff distances.

The algorithm above is needed because standard GF design only explores the nutritional space through rails, which are lines that subdivide the nutrient space. This means that a large portion of the space remains unexplored, and the approach above is needed to cover these unexplored spaces as shown in the second study of the Nutrigonometry series (see [40]). A full coverage of the nutrient space is needed for a global analysis of the properties of the performance landscapes (see also ‘Discussion’ section for more on this topic).

### 3.2. Difference between performance landscapes

We used the Hausdorff distance *d*_*H*_(*S*_*m*_, *S*_*n*_) to estimate the difference between two performance landscapes *S*_*m*_ and *S*_*n*_. Note that the two performance landscapes can be of two traits or the landscape of a trait and the flat landscape with same domain. We used the Hausdorff distance because it measures the distance between two sets of non-empty compact (‘closed’) subsets of a given metric space and thus provide a way to measure the overall differences between two subsets. For three-dimensional performance landscapes, the metric space is $R^{3}$, and the non-empty subset within the metric space are the performance landscapes *S*_*m*_ and *S*_*n*_. Formally, the Hausdorff distance *d*_*H*_(*S*_*m*_, *S*_*n*_) is defined as

3.1 $$d_{H}(S_{m},S_{n})=\text{inf}\{\epsilon \geq 0;S_{m}\subseteq S_{n_{\epsilon }}\;\text{and}\;S_{n}\subseteq S_{m_{\epsilon }}\}$$

whereby ϵ is the distance which is necessary for the subset *S*_*m*_ to contain the subset *S*_*n*_ and vice versa (note that the definition is symmetric). One can think of ϵ as the smallest quantity needed to expand the subset *S*_*m*_ such that it contains set *S*_*n*_ and subset *S*_*n*_ to contain *S*_*m*_. The bidirectionality of the definition of the Hausdorff distance ensures that the value of *d*_*H*_(*S*_*m*_, *S*_*n*_) equals zero if and only if the two sets (or in this case, the two landscapes) are the same. This implies that the distance between two sets may differ depending on the way the calculation is conducted. For example, consider two sets *A* = {1, 2} and *B* = {1, 2, 3, 4}. If the Hausdorff distance was unidirectional, then the distance between sets *A* and *B* would equal zero, since *A*⊆ *B* (i.e. the numbers 1 and 2 in set *A* are also present in set *B*). Now, considering the bidirectionality of the definition of Hausdorff distance given above, we also need to calculate the distance between set *B* and set *A*, which is equal to 2 (i.e. the maximum distance between the elements of *B* and *A* so that *B*⊆ *A* is 4_*B*_ − 2_*A*_ = 3_*B*_ − 1_*A*_ = 2, where the subscripts represent the sets from which the number belongs in this example). Thus, performance landscapes that are identical have Hausdorff distances *d*_*H*_(*S*_*m*_, *S*_*n*_) equal to zero. To avoid scale effects when estimating ϵ, we mean-standardized the *z*-axis (i.e. divided by the variables’ mean) to ensure that the estimates of *d*_*H*_(*S*_*m*_, *S*_*n*_) were obtained on the same scale for two performance landscapes.

### 3.3. Datasets and model application

To demonstrate the functionality of our model we first implemented the model in two canonical datasets: a flat surface (*x*, *y*, 1) and a saddle surface (*x*, *y*, *xy*). Next, we applied the model to empirical performance landscapes from a landmark GF dataset, to estimate properties of the performance landscapes for lifespan, daily egg production (daily eggs) and lifetime egg production in *D. melanogaster* [6]. Gauss and mean curvatures, as well as the surface-area ratio of the canonical and empirical performance landscapes, were calculated as explained in the previous section (see ‘The Model’ section).

## 4. Results

### 4.1. Evaluating model performance for landscape construction

**Figure 2.**
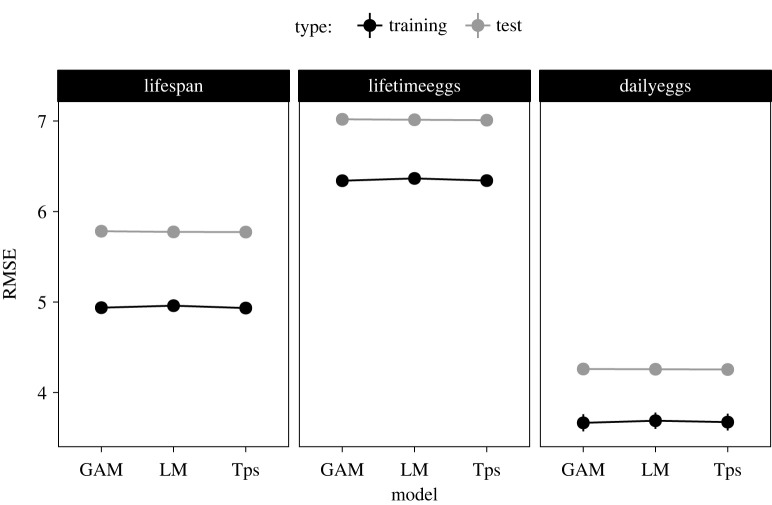
Performance of commonly used models to analyse GF data. RMSE, root-mean-square-error; Training, training dataset; Test, testing dataset.

The fundamental assumption for our model is that performance landscapes could be approximated using a polynomial regression model of the form *ax*^2^ + *by*^2^ + *cx* + *dy* + *exy*. Yet, in GF studies, other models are commonly used to analyse the data, such as GAMs and TPS. These models cannot be represented with closed quadratic polynomial equations but could result in better fit of the data and consequently, better approximations of the performance landscapes. To test this, we compared the model performance (using RMSE) of the quadratic polynomial linear regression (LM), GAM and TPS models across the three traits of the dataset. The results show that the LM model has similar performance in both training and test datasets for all traits (figure 2). Therefore, quadratic LM models are a valid approximation for analysing GF data. More importantly, this enables us to represent the regression model in the quadratic polynomial form, which facilitates the calculations of curvature and surface-area ratios.

### 4.2. Model application to canonical datasets

We first demonstrate the use of our model by applying it to canonical datasets. We calculated the surface-area ratio of the canonical landscapes, where we expected the ratio to equal 1 for the *flat* landscapes (as a flat landscape has the same area as itself) and greater than 1 for the *saddle* landscape, due to the additional surface-area resulting from its curvature. Indeed, surface-area ratio of the the *flat* landscape was equal to 1 while the surface-area ratio for the *saddle* landscape was equal to 3.982, revealing that the *saddle* landscape had approximately four times more surface-area than a flat landscape with the same domain. This shows that the saddle canonical landscape used here was *ca* four-fold more wiggly than a flat landscape. Importantly, aside from rounding error, this corroborates analytical expectation of the ratio between a flat landscape and a saddle landscape given by $\int_{0}^{1}\int_{0}^{1}xy\;\text{d}x\;\text{d}y/\int_{0}^{1}\int_{0}^{1}1\;\text{d}x\;\text{d}y=4$.

**Figure 3.**
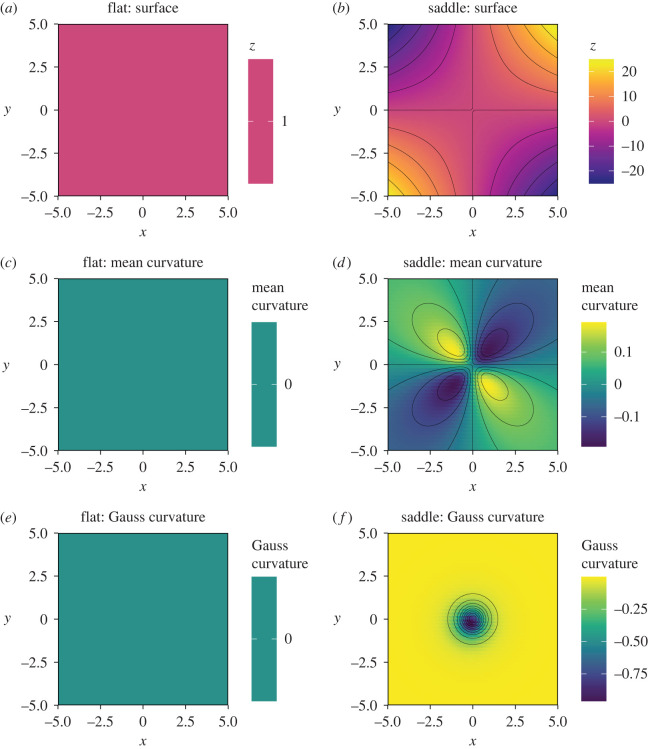
Gauss and mean curvatures of two canonical datasets. (*a*) Simulated flat and (*b*) saddle landscapes. (*b*,*c*) Mean curvatures of the flat (*c*,*d*) saddle landscapes, respectively. (*e*,*f*) Gauss and mean curvatures of (*e*) the flat and (*f*) the saddle landscapes, respectively.

Next, we calculated curvature. By definition, *flat* landscapes do not have curvature and, as expected, we found that the model gives both Gauss and mean curvatures equal to zero for all points (figure 3). Conversely, for the *saddle* landscape we expected a point in which the landscape resembles a sphere at (*x*, *y*) = (0, 0). At this point, the Gauss curvature is expected to be −1 and mean curvature, 0, provided that principal curvatures are equal to 1 and −1 [32]. Mean curvature, on the other hand, was expected to be positive for regions in the saddle where the landscape bends downwards and negative for regions that bend upwards. This prediction emerges from theory on the behaviour of the normal unit vector $\vec{n}$ in saddle regions [32]. As expected, our model found that both Gauss and mean curvatures were zero for a *flat* landscape. Moreover, the model found that Gauss curvature equals −1 at the point (*x*, *y*) = (0, 0) in the *saddle* landscape, and that mean curvature was positive and negative for regions bending downwards and upwards, respectively (figure 3). Together, these results demonstrate that our model can accurately estimate the curvature and surface-area ratio as a proxy of landscape wiggliness in canonical datasets.

### 4.3. Model application to empirical datasets

Next, we applied the model to an empirical landmark GF dataset to estimate curvature and surface-area (see [6]). As in the previous section, we first estimated the surface-area ratio for the performance landscape for the three performance traits measured in the dataset: lifespan, lifetime egg production (lifetime eggs) and daily egg production (daily eggs). Our model found that the performance landscape for lifespan had the largest deviations from a flat landscape (*surface-area ratio:* 51.125), followed by the the performance landscape of lifetime eggs (*surface-area ratio:* 49.587) and daily eggs (*surface-area ratio:* 4.194), respectively. These differences in the overall conformation of the performance landscapes were corroborated by the estimates of Hausdorff distances *d*_*H*_(*S*_*m*_, *S*_*n*_). All performance landscapes differed significantly from flat, but the magnitude of this difference was greater for lifespan, lifetime eggs and daily eggs, respectively. This suggests that the performance lanscape for lifespan is the most different from a flat landscape, while the performance landscape for daily eggs is the closest (relatively speaking). We also compared the differences between landscapes using the Hausdorff distances and found that, as expected, the landscapes from lifespan and daily eggs are the most different, followed by the landscapes of lifespan and lifetime eggs and lifetime eggs and daily eggs, respectively (table 1).

**Table 1.** Hausdorff distance estimates for the performance landscapes.

	*d*_*H*_(*S*_*m*_, *S*_*n*_)			
comparison	mean	s.d.	lwr 95% CI	upr 95% CI
lifespan–flat	2.411	0.270	1.426	2.534
lifetime eggs–flat	1.940	0.110	1.765	2.186
daily eggs–flat	1.783	0.134	1.375	1.967
lifespan–lifetime eggs	2.118	0.177	1.616	2.315
lifespan–daily eggs	2.615	0.197	2.589	3.311
lifetime eggs–daily eggs	1.661	0.212	1.418	2.214

**Figure 4.**
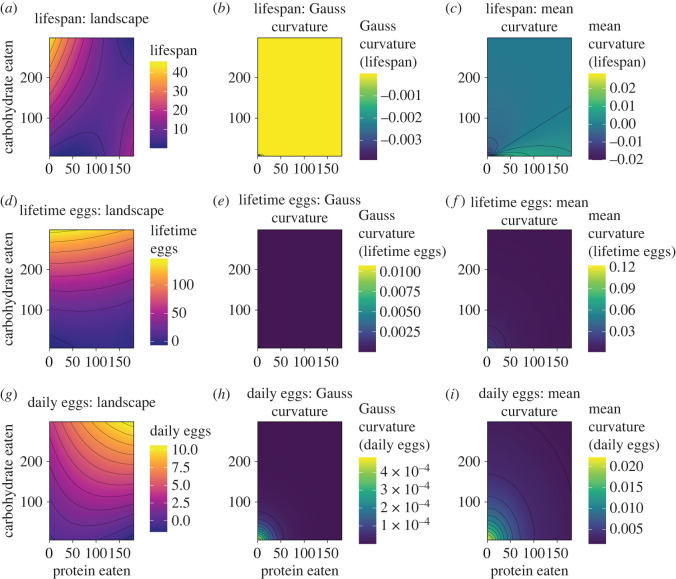
Gauss and mean curvatures of performance traits. (*a*–*c*) Landscape (*a*), Gauss curvature (*b*) and mean curvature (*c*) for lifespan. (*d*,*f*) Landscape (*d*), Gauss curvature (*e*) and mean curvature (*f*) for lifetime egg production. (*g*–*i*) Landscape (*g*), Gauss curvature (*h*) and mean curvature (*i*) for daily egg production (daily eggs).

We then investigated the curvature of the performance landscapes. Overall, the majority of the landscape had zero Gauss and mean curvatures, suggesting that these regions were equivalent to flat inclined planes. For instance, lifespan, which was the landscape with the greatest surface-area, had small regions of negative Gauss curvature and positive mean curvature at low nutrient intakes (i.e. closer to the origin), which disappeared for other regions of the landscape (see figure 4*a*–*c*). Likewise, the performance landscape for lifetime egg had virtually zero Gauss and mean curvatures throughout, suggesting that the landscape as a whole was an inclined plane (see figure 4*d*–*f*). The performance landscape for daily egg production was the only landscape that showed higher curvature estimates in regions close to the origin (i.e. low nutrient intake) (see figure 4*g*–*h*). These results suggest that the performance landscape for daily eggs, which responds strongly to the interaction between protein and carbohydrate intakes, had relatively more positive curvature in the region of low nutrient intake. This is important because it can suggest that, in regions of low nutrient intake, animals can pay larger costs from small nutritional imbalances. This can be interpreted as analogous to walking along a ridge: small deviations from the path have potentially large implications for the position in the ridge (e.g. falling either side of the crest). Overall though, the results show that estimating surface-area and curvature of performance landscapes can reveal important properties of the landscapes with potential biological significance (see Discussion for hypothesis generated from curvature).

## 5. Discussion

We proposed a new model to measure properties of performance landscapes related to surface-area and curvature. Moreover, we introduced the Hausdorff distance which can be used to compare the similarity between two *n*-dimensional performance landscapes. Our analysis first corroborates the underlying assumption that performance landscape could be approximated by a quadratic polynomial of the form *ax*^2^ + *by*^2^ + *cx* + *dy* + *exy*. In fact, we showed that this polynomial fits the data as well as other commonly used models to analyse and interpolate GF data (i.e. GAM and TPS). These approximations are useful because *exact* curves and landscapes are never accessible in real-world biological data due to, for example, sampling limitations and variability in responses. Therefore, the approximations used here and in previous studies provide a way in which landscapes can be constructed from a relatively sparse grid of known biological responses [19]. We used canonical simulated datasets and an empirical dataset from [6], which measured the performance landscapes for lifespan and daily eggs, to demonstrate the application of our model. Together, the model proposed here is the first to estimate properties of performance landscapes other than peaks and valleys. This enables new insights from studies of nutrition in high dimensions. Currently, we do not know how (or whether) animals ‘navigate’ performance landscapes in similar ways to fitness landscapes (e.g. [30]). Recent studies integrating the concept of performance landscapes and genetics have been carried out (see [41]) and simulations have provided insights into the genetic variance in response to diet for lifespan [42]. This remains a subject of future theoretical development. Despite this, from the perspective of curvature of multiple performance landscapes, one question emerges: how are the different performance landscapes integrated so that they influence animal nutritional resource allocation based on nutritional trade-offs? In other words, how do animals allocate nutrients to competing resources with different performance landscapes? The curvatures of the performance landscapes, if important, suggest that the cost-benefit functions are nonlinear for different traits. Therefore, animals have to allocate resources to competing traits taking into account the nonlinearity of the performance landscapes of each trait *i*, for all traits. More formally, we can state that for all fitness traits *w* with landscape *S*_*w*_, animals will evolve to allocate resources such that the animal optimizes some weighted average of all performance landscapes *S*_*w*_. This implies that animal nutrition is in Pareto optimality, whereby any deviations on the nutrient intake or allocation to a trait *w*_*i*_ would result in a cost to other traits *w*_*j*_ [43]. Pareto optimality in nutritional decisions has been demonstrated in computer algorithms [44,45] but to our knowledge, has not yet been shown in empirical data. Currently, we rely on the growing use of GF to generate landscapes to test Pareto optimality on nutritional trade-offs and feeding behaviour. A formal derivation of the Pareto optimal model is outside the scope of this paper and will be the subject of future work.

Previous analytical models using GF data focused on identifying peaks and valleys in performance landscapes in order to estimate the extent to which animals had to compromise in their nutrition for optimal trait expression. For instance, [26] proposed a model which relied on slopes of linear regressions as components of a vector to compare the strength of nutritional trade-offs between traits, estimated as the angle *θ* between vectors. Likewise, Morimoto & Lihoreau [27] used a similar idea but instead of slopes they used the position coordinates of the regions of interest (i.e.peaks or valleys) as components of a vector and estimation of *θ*. The coordinates for the region of interest was found using machine learning support-vector-machine (SVM) with radial kernel. del Castillo *et al.* [28] used bootstrapping approaches to identify the region within the performance landscape that represented the landscape optimal, which could then be compared with the similar region in other traits using confidence regions generated by the method (see also [41]). This approach had the advantage of not relying on any particular parametric distribution for generating estimates. More recently, Pascacio-Villafán *et al.* [25] implemented a standard optimization algorithm from response surface modelling approaches to identify and compare regions of interest in the landscape, primarily focusing on the comparison between peak regions. While these previous analytical models are useful, they have focused on identifying and comparing either peaks or valleys in performance landscapes, an approach that might have overlooked other properties of the landscapes which can contain biologically relevant information. The model proposed here addresses this limitation, as it estimates curvature properties from the performance landscapes. Importantly, we showed how the framework can be used to estimate surface-area and curvature of performance landscapes, which can aid interpretation and generate important predictions of animal responses to nutrition. For instance, as explained above, surface-area can be an important indicator of wiggliness in performance landscapes, as landscapes with more oscillations also have higher surface-area relative to flat landscapes (e.g. increased surface-area of cells due to microvilli). By estimating surface-area, it might be possible to predict how animals might respond physiologically and behaviourally to various nutrient imbalances. Similarly, curvature (both Gaussian and mean curvatures) can also enable further characterization and prediction of responses to nutritional imbalances. Landscapes with regions of high (or low) curvature can lead to terrains in which animals are expected to experience higher costs of nutrient imbalances than regions in landscapes with low curvature (e.g. inclined plane). In our analysis of the empirical dataset of life-histories in *D. melanogaster*, we found that only the landscape of daily egg production showed relatively high local curvature at low nutrient intakes. This coincided with the valley region in the performance landscape, and confirms the nonlinear costs of low nutrient intake in the expression of this trait. More empirical studies as well as more data for performance landscapes are needed to develop a more intuitive relationship between analytical properties of the performance landscapes (e.g. local curvature) and the broader patterns of animal nutrition [46]. Nevertheless, we showed that our approach can be a powerful ally to characterize properties of performance landscapes that can aid biological insight.

Studies using GF have used a range of standard statistical models to analyse the data, but to date, the performance of these models had not been properly scrutinized. In particular, LMs, GAM and TPS have been the most commonly used approaches in GF studies (see e.g. [6–9,12,13,23,24,47–49], and references therein) (see also [50]) for cubic splines. In this study, we had to assume that performance landscapes could be approximated using a general LM of the form *ax*^2^ + *by*^2^ + *cx* + *dy* + *exy* in order to calculate surface-area and curvature. We tested this assumption by measuring the performance of LM, GAM and TPS onto GF data, and showed that indeed, an LM can be as good a model to GF data as the more complex GAM and TPS. This is important because the equation *ax*^2^ + *by*^2^ + *cx* + *dy* + *exy* is differentiable and enables easy calculations of surface integrals and gradients for the estimates of curvatures. We have recently analysed the performance of several statistical (machine learning) models and their performance in identifying peak and valley regions in performance landscapes [29]. The good quadratic approximation using LM presented in this study agrees with our extensive comparison of model performance in GF datasets. Therefore, the results suggest that LMs are, in principle, reasonable approximations for the analysis of GF datasets for characterization of regions in the performance landscape. With the growing use of GF in nutritional studies, it is crucial that the raw data to construct the performance landscapes are made open access as this will enable us to test whether the quadratic approximations presented here are suitable for other performance traits and species.

It is important to mention that the model proposed here, and all previous models developed in the literature which rely on—or estimate properties from—performance landscape assume that the landscape itself can be estimated accurately. This may not necessarily be the case for a standard GF design which relies on nutritional rails and explores a subset of all possible regions in space, leaving large parts of the space unexplored (particularly in regions that correspond to the interactions between nutrients, that is, along the diagonal of the nutrient space) [40]. The rationale for empirically testing a subset of diets and hence, regions of the nutrient space, is that results are reliable only if ecologically relevant ranges of diets are tested (see Point 4 in [51]). More studies are needed to understand the consequences of imbalanced diets beyond those in which animals have evolved. We argue that exploring more combinations of diets—and hence, regions of the space—can enable more accurate approximations of performance landscapes and consequently, better predictions of landscape properties. This may come with higher practical cost (e.g. higher number of experimental diets needed) which is justified if better performance landscapes are generated as outputs. Optimal experimental designs in nutritional studies is an active research area [52] (see also [4,18,19,50]).

In conclusion, we proposed a novel model to measure curvature properties of performance landscapes using the GF for nutrition. The model estimates surface-area and curvature of performance landscapes and, for the first time, estimates properties of performance landscapes other than peaks and valleys. This opens up the range of parameters that can be estimated from performance landscapes, which may prove important for biological insights. We also introduce the use of the Hausdorff distance as a metric for pairwise comparison of performance landscapes. The methodology proposed here can be employed to landscapes in general, for comparisons within and between species (for any fitness-related trait) and thus, can play an important role in our understanding of the responses to nutrition across the animal kingdom [5].

## Data Availability

The data used in the paper is available from the Dryad Digital Repository: https://doi.org/10.5061/dryad.tp7519s [53]. R code is available as an R Markdown electronic supplementary material [54].
